# Short impact on soil microbiome of a *Bacillus amyloliquefaciens* QST713 based product that correlates with higher potato yield across USA

**DOI:** 10.3389/fpls.2024.1332840

**Published:** 2024-03-12

**Authors:** Irene Adamo, Marta Acin-Albiac, Sam Röttjers, Diego Rodríguez de Prado, Blas M. Benito, Jorge Zamora, Rakesh Godara, Beatriz García-Jiménez, Panpan Jiang-Rempel, Lauren C. Cline, Alberto Acedo

**Affiliations:** ^1^ Data Science, Biome Makers Inc, Davis, CA, United States; ^2^ IT/Bioengineering Biome Makers Inc, Davis, CA, United States; ^3^ Bayer Crop Science, Monheim, Germany; ^4^ Bayer Crop Science, St. Louis, MO, United States; ^5^ CSO, Biome Makers Inc, Davis, CA, United States

**Keywords:** biostimulant, soil microbiome, potato yield, potato leaf petiole, potato peel, network properties

## Abstract

Potato (*Solanum tuberosum L.*) is considered one of the most widely consumed crops worldwide, due to its high yield and nutritional profile, climate change-related environmental threats and increasing food demand. This scenario highlights the need of sustainable agricultural practices to enhance potato productivity, while preserving and maintaining soil health. Plant growth-promoting bacteria (PGPB) stimulate crop production through biofertilization mechanisms with low environmental impact. For instance, PGPB promote biological nitrogen fixation, phosphate solubilization, production of phytohormones, and biocontrol processes. Hence, these microbes provide a promising solution for more productive and sustainable agriculture. In this study, the effects of *Bacillus amyloliquefaciens* QST713 based-product (MINUET™, Bayer) were assessed in terms of yield, soil microbiome, potato peel and petiole nutrient profile as a promising PGPB in a wide range of potato cultivars across the United States of America. Depending on the location, potato yield and boron petiole content increased after biostimulant inoculation to maximum of 24% and 14%, respectively. Similarly, nutrient profile in potato peel was greatly improved depending on the location with a maximum of 73%, 62% and 36% for manganese, zinc and phosphorus. Notably, fungal composition was shifted in the treated group. Yield showed strong associations with specific microbial taxa, such as *Pseudoarthrobacter, Ammoniphilus, Ideonella, Candidatus Berkiella, Dongia*. Moreover, local networks strongly associated with yield, highlighting the important role of the native soil microbiome structure in indirectly maintaining soil health. Our results showed that treatment with *B. amyloliquefaciens* based product correlated with enhanced yield, with minor impacts on the soil microbiome diversity. Further studies are suggested to disentangle the underlying mechanisms of identified patterns and associations.

## 1 Introduction

Potato (*Solanum tuberosum L.*) is one of the most widely consumed crops worldwide. Because of its high yield and nutritive values, it is considered one of the most important agricultural crops (Beals, 2019; Kanter and Elkin, 2019), whose production reached a total 376 million tonnes in 2021 as per Unicef organization (FAO, 2022). However, current environmental threats to the agricultural sector related to climate change, biodiversity loss, land degradation and agricultural practices raised interest in more sustainable agricultural practices to preserve and maintain soil health (Muller et al., 2017). In addition, the total global food demand is expected to increase by 35% - 56% between 2010 and 2050 (Van Dijk et al., 2021). Therefore, an intensified effort to enhance food production is needed to overcome these global future challenges, while reducing the environmental impact on the ecosystems (Rouphael and Colla, 2020). Plant growth-promoting (PGP) microbes are known for their capacity to stimulate crop production through biofertilization mechanisms with low environmental impacts. For instance, PGP microbes promote biological nitrogen fixation, phosphate solubilization, production of phytohormones, and biocontrol processes (Vacheron et al., 2013; Bashan et al., 2014; Shahrajabian et al., 2023). More in particular, plant growth-promoting rhizobacteria (PGPR) suggest a promising solution for more productive and sustainable agriculture practices (Chaudhary et al., 2023). The use of biological products in agriculture has increased substantially in the last decade. In 2022, the plant-growth promoters and biostimulants market was valued 2.9 billion and ca. USD 3.5 billion, respectively (Markets and Markets, 2022).

Several studies have already assessed the positive direct effects of PGPR on potato yield (Sarwar et al., 2018; Ekin, 2019; Imam et al., 2021). Therefore, microbial biostimulants are an innovative and promising group of agricultural inputs. Nevertheless, more effort is needed to explore their use and the possible positive impacts on productivity and soil health in potato cultivars. This may provide inputs to give recommendations to farmers on the use of more sustainable practices (Shahrajabian et al., 2023). Because of the importance of soil microbial communities in maintaining soil quality and global nutrient cycling, the effects of plant growth-promoting bacteria soil inoculation should be further assessed (Bharti et al., 2016; Imam et al., 2021; Chaudhary et al., 2023).

Moreover, studies at global and local levels observed that soil microbial communities are affected by changes in soil physio chemistry and climate (Tedersoo et al., 2014; Glassman et al., 2017; Plassart et al., 2019; Zeng et al., 2019). However, associations between soil physio chemistry and soil microbiome before and after product inoculation are still poorly understood. In addition, potato petiole analysis can give indirect information on the effectiveness of products by monitoring nutrient levels in potato cultivars (Collins et al., 2016; Kong et al., 2022). For instance, phosphorus, boron, nitrogen, zinc and manganese concentrations increased upon rhizobacteria inoculation in strawberry and wheat crops (Ipek et al., 2014; Kumar et al., 2014). Nevertheless, still little is known about to what extent potato petiole nutrients are important to assess the efficacy of biological products and how it can relate to potato yield and soil microbiome.

Similarly, a previous study analyzed the microbial composition and structure of bulk and rhizosphere soils to assess the effects of *B. amyloliquefaciens* QST713-based product (Imam et al., 2021). Imam et al. (2021) investigated the effects of this biological product in three different geographical regions in the United States. Their results showed that the product positively promoted potato yield in two geographic locations. Moreover, microbial taxa abundances and community structure changed after inoculation, but long-lasting effects on soil microbial alpha and beta-diversity were not observed. Finally, yield prediction model including all three locations was built, which incorporated product use and soil microbiome information, such as microbiome network properties. These properties define how microorganisms tend to co-occur or co-exclude and measure how the microbial network tends to cluster together in a specific ecological niche. Imam et al. (2021) concluded that soil fungal network properties were the most important predictors of yield.

In our study, the effects of *B. amyloliquefaciens* QST713 application were addressed in a wide range potato cultivars across the United States of America, in 21 geographic locations. This product has already demonstrated a broad fungicide and bactericide activity on potato crops (US Environmental Protection Agency, 2006). Moreover, its antibiotic production and *in vitro* suppression of pathogens were examined from root surfaces through HPLC and MS quantification (Kinsella et al., 2009). In this study, previous work of Imam et al. (2021) was extended by exploring the possible effects of *B. amyloliquefaciens* based product on a substantially broader geographic distribution. Here, product effects on soil microbiome composition and structure were determined, comparing both control and treated samples at growth stage 2 (around 30 days after emergence) with control before planting. Moreover, crop yield, soil physicochemical properties, potato peel and leaf nutrients, along with environmental data, were integrated to explore the effetcs of *B. amyloliquefaciens* QST713-based product on soil microbiome associated with crop performance in different locations. Indeed, this study demonstrated the impacts of this biological product on soil microbiome and potato productivity. Finally, the results provided insights on the functioning of soil ecosystems and its associations with crop performance. This may serve to provide better recommendations to growers on the use of more sustainable products in the current global change context.

## 2 Materials and methods

### 2.1 Agronomic trial design and sample collection

Potatoes (Varieties: Russet Ranger, Russet Burbank, French Fingerling, Russet Norkotah, FL1867, FL2137, Red Norland) were planted between April and May 2020 in 21 different geographical regions in the United States (**Supplementary Figure S1**; **Supplementary Table S1**). All trials were managed by Bayer Crop Science. Treatment consisted of a biological product containing minimum of 2.7 x 10^10^ colony forming units (CFU) of *B. amyloliquefaciens* strain QST713 (NCBI accession number: CP025079; MINUET™, Bayer Crop Science). All trials comprised three replicated sub-plots per treatment condition for a total of 6 sub-plots per location. Harvest was conducted by corresponding Bayer trial cooperators located in Idaho, Washington, Texas, Michigan, Maine, New York, North Dakota, Wisconsin, Colorado and Nebraska. Potato tubers were sampled at harvest and yields were evaluated and recorded in pounds and hundredweight per acre (cwt/ac). Soil samples were collected at two different time points: before planting (T0) and at growth stage 2, around 30 days after emergence (T1) of each variety. In addition, each variety had different time frame duration from time points T0 and T1, depending on their growth speed. Therefore, potato variety influence was mitigated. Detailed sample collection dates can be found in **Supplementary Table S1**. To encompass the variability of field, bulk soil core samples were collected from sub-plots to form a well-blended composite soil sample, using 1 inch diameter soil probe (Hartz, 2007). For each time point, samples were collected from both control and treated samples to isolate the effect of the treatment. For T0, a total of 132 samples were collected (66 control 66 treated), while for T1 a total of 130 samples (65 control 65 treated).

### 2.2 Weather measurements

Weather variables were downloaded from several public databases such as Bioclim (Karger et al., 2017), Long-term Moderate-Resolution Imaging Spectroradiometer (MODIS) - Land Surface Temperature (LST) day-time and night-time temperatures, sd and differences at 1 km based on the 2000–2017 time series (Hengl et al., 2017). Moreover, soil variables were downloaded from Copernicus Global Land services (https://land.copernicus.eu/global/index.html) and International Soil Reference and Information Centre (ISRIC) World Soil Information (Hengl et al., 2017). Then, a correlogram was constructed to check and reduce for collinearity between variables. Feature selection was performed based on Principal Component Analysis (PCA). Then, the number of dimensions that explained a percentage of variance between 90-95% and the variables that most contributed to each selected dimension were selected: Monthly median soil temperature during day time based on data from the MODIS sensor; average normalized difference vegetation index (NDVI) of the first third of April (m04) between 1999 and 2019; mean temperature of wettest quarter, temperature seasonality (standard deviation ×100); precipitation of the coldest quarter of the year, soil sand percent in the first 30 cm of soil, soil clay percent in the first 30 cm of soil and average NDVI of the first third of July (m07) between 1999 and 2019 were used. Full description of selected environmental features can be found in **Supplementary Table S2**.

### 2.3 Soil physicochemical, leaf petiole and potato peel quantification

A total of 120 observations were taken for soil physicochemical properties at time point T0, while a total of 112 observations were taken for petiole at time point T1, and 98 observations for yield at harvest. Mean and standard deviation of each soil property in each location can be found in **Supplementary Table S3**. Leaf petioles were collected from the last mature leaf of potato plants, all leaf tissues were removed. Potato peel consisted of 216 observations taken at harvest. Soil and leaf petiole chemistry were analyzed by Ward Laboratories Inc (Nebraska, United States) with common analytical methods (https://www.wardlab.com/services/plant-analysis/). Potato peel metabolomics were analyzed by Bayer AG. Briefly, potatoes were washed and peeled off for weighing. Then, potato peels were put into liquid nitrogen for metabolomics. Lastly, metabolomics quantification was done using mass spectrometry. Soil physicochemical properties in parts per million (ppm) included nitrate, potassium, sulfur, zinc, manganese, copper, calcium, phosphorus. Soil physicochemical properties in weight percentage: organic matter (LOI), sand, silt and clay. Petiole nutrients in weight percentage included nitrogen, phosphorus, potassium, magnesium and calcium, while nutrients in ppm included zinc, iron, copper and boron. Finally, potato peel nutrients in unit percent included total nitrogen, phosphorus, potassium, calcium, magnesium, sulfur, sodium, while nutrients in ppm included zinc, iron, copper and boron.

### 2.4 DNA extraction and library preparation

After collection, soil samples were immediately sent for molecular analysis to the Biome Makers laboratory in Sacramento, CA. DNA extraction was performed with the DNeasy PowerLyzer PowerSoil kit from Qiagen. To characterize both bacterial and fungal microbial communities associated with bulk soils, BeCrop® custom primers were used for PCR amplification, specifically targeting the 16S rRNA V4 region and the ITS1 region (Becares and Fernandez, 2017). Next, amplicons were purified using the KAPA Pure Beads (Roche) kit, while correct 16S and ITS amplification was assessed through agarose gel. Purified PCR products were then subjected to library preparation, following a two-step PCR Illumina protocol (Gobbi et al., 2019; Liao et al., 2019). Next, DNA was quantified using a Qubit fluorometer with Qubit HS Assay Kit 500 (Thermo Fisher Scientific). Finally, libraries were sequenced on an Illumina MiSeq instrument (Illumina, San Diego, CA, USA) using 2×251 paired-end reads.

### 2.5 Bioinformatic processing

Primers were removed from paired end reads using Cutadapt (Martin, 2011). Then, the trimmed reads were merged with a minimum overlapping of 100 nucleotides. Next, the sequences were quality filtered by Expected Error with a maximum value of 1.0 (Edgar et al., 2011). After quality pre-processing, reads having single nucleotide differences were iteratively clustered together to form ASVs (Amplicon Sequencing Variants) using Swarm (Mahé et al., 2022). *De novo* chimeras and remaining singletons were subsequently removed (Edgar et al., 2011). Finally, taxonomy was assigned from ASVs using a global alignment with 97% identity, against a curated reference database from SILVA 138.1 for 16S sequences, and UNITE 8.3 for ITS sequences (Glöckner et al., 2017; Nilsson et al., 2019).

### 2.6 Computation of local network properties

Local network properties were determined following the procedure described by (Ortiz-Álvarez et al., 2021). Briefly, microbial community networks were built for 16S and ITS samples independently following the methodology described by Veech (2013). Presence-absence metanetwork with all samples was built using rarefied counts, and the ASV pairs occurring significantly more or fewer than expected by chance were preserved to create the co-occurrence or co-exclusion network, respectively. Local network properties were retrieved from subsetting ASV pairs from the corresponding metanetwork present in the individual sample. The following network properties were computed for both co-occurrence and co-exclusion in 16S and ITS: modularity, transitivity and average path length. Modularity defines microorganisms that tend to co-occur or co-exclude frequently in specific ecological niches (clusters). Next, transitivity (clustering coefficient) measures the degree to which nodes in a network tend to cluster together. Finally, average path length quantifies the degree of connectivity to go from one side of the network to another.

### 2.7 Computation of global networks integrating yield and leaf physiochemical properties

The ASV data were aggregated to genus-level and rarefied to 10.000 reads for 16S, 18.750 for ITS. Five samples had insufficient reads and were removed. Here, both treated and untreated samples available were used. Then, treatment was introduced as a metadata variable to assess associations to MINUET™ biological fungicide or control treatment. A prevalence filter of 20% was then applied to the genera. Throughout all steps, the total reads per sample were preserved, with the final data set containing 63 genera across 39 samples for the 16S data and 197 genera across 44 samples for the ITS data. Next, a permutation test was performed to identify taxa with higher or lower degree than expected given their prevalence in the network. Yield, petiole and environmental data were centered and scaled and used in network inference, while location and treatment were used as one-hot encoded variables. Nodes that represent these metadata variables were referred to as MVs. FlashWeave v0.18.1 was run to assess direct associations in Julia version 1.7.1, with heterogeneous set to false and sensitive to true (Tackmann et al., 2019).

### 2.8 Statistical analysis

Statistical analyses of microbiome data were done mainly using phyloseq, microbiome and vegan R packages (McMurdie and Holmes, 2013; Oksanen, 2013; Shetty and Lahti, 2019). First, the generation of rarefaction curves allowed estimating sample intradiversity in terms of bacterial and fungal richness and Shannon index at the same sequencing depth. Then, pairwise comparison of treated vs control samples for microbiome indexes (biodiversity and network properties), yield, petiole and potato quality were performed through Wilcox-test using Z-score transformation, standardized by location. Moreover, correlations between microbiome indexes (network properties and biodiversity), yield, soil physico-chemical, petiole nutrients, potato peel nutrients and environmental variables at time T0 and T1 were performed through Spearman correlation. Resulting p-values were further corrected by False Discovery Rate (FDR). Then, significant correlations were visualized as a network graph. Here, Louvain clustering was performed to identify clusters of positive associations among variables. Microbiome composition was assessed through beta diversity analysis, using Principal Coordinate Analysis (PCoA) ordination and Bray-Curtis distance matrix. Then, explained variance of resulting ordination by treatment, time and location and their interaction was determined through PERMANOVA. In addition, soil, petiole, potato quality physico-chemical properties and environmental properties were correlated to bacterial and fungal ordination, through the envfit. In addition, constrained distance-based Redundancy Analyses (RDAs) were generated using log-transformed data of microbial counts. Next, microbiome data was centered based on the location and fitted to a linear model containing both time and treatment, along with potato peel, petiole and soil physiochemical properties and weather variables. Significance of independent variables was determined through ANOVA. Only significant variables were visualized in the ordinations. Then, yield, petiole and environmental data were centered and scaled and used in the global network inference, while location and treatment were used as one-hot encoded variables. Networks were visualized in Cytoscape 3.9.0. Prevalence of conserved soil prokaryotic and fungal genera was assessed in control and treated samples at T0 and treated samples at T1. This was visualized as heatmaps at varying detection thresholds. Then, shared taxa at ASV and genus level among control and treated samples at time T0 and time T1 was visualized through Venn diagrams with constrained intersections by location. Finally, differential abundant (DA) taxa due to product application were determined through negative binomial regression at various taxonomic levels (Love et al., 2014).

## 3 Results

### 3.1 Application of the *B. amyloliquefaciens* based product increased yield, leaf petiole boron, potato peel manganese, phosphorus and zinc content.

**Figure 1:**
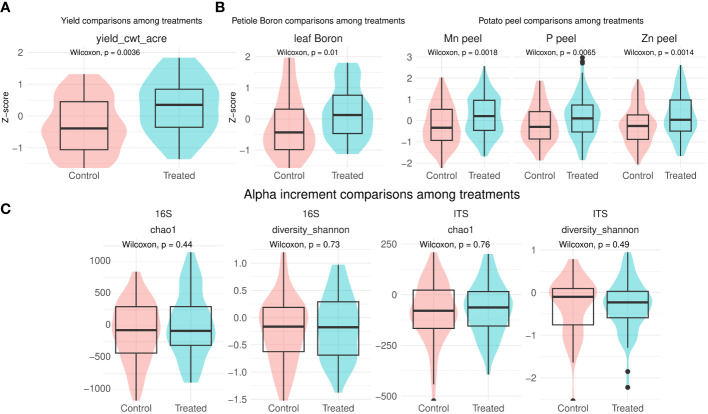
Yield **(A)**, leaf boron and and potato peel **(B)** comparisons between treatment and control normalized per location. Only significant comparisons are shown. Net change in biodiversity from T0 to T1 comparison between treatment and control **(C)**. Complete comparisons of leaf petiole and potato peel nutrients can be found in **Supplementary Figure S1**.

Yield significantly increased in treated sample, when standardizing by location (Wilcoxon-test: p-value = 0.003, **Figure 1A**). For individual locations, a similar trend was observed, with a percentage of increment between 24 and 2% in almost all locations (**Supplementary Figure S2**). However, no statistical significant differences in treated vs control were seen per location. Conversely, yield showed significant differences across locations (Kruskal-Wallis: p-value < 0.001); with, Loc12 and Loc 15 resulting in the highest and lowest yield, respectively (**Supplementary Figure S3**).

Leaf petiole boron was also significantly enhanced in treated samples, when standardised by location (increment across locations: 14 - 2%, Wilcoxon-test: p-value = 0.01, (**Figure 1A**). Similarly, *B. amyloliquefaciens* significantly enhanced potato peel manganese, phosphorus and zinc content (increment across locations: 73 - 2%, 36 - 2%, 62 - 2%, respectively, Wilcoxon-test: p-value = 0.007, p-value = 0.002, p-value = 0.001, **Figure 1B**).

Bacterial and fungal richness and evenness significantly differed across locations independently of time point, as shown by Chao1 and Shannon indexes, respectively (**Supplementary Figure S4**). Metadata together with computed alpha diversity per sample are found in **Supplementary Table S1**. No significant differences were detected for either bacterial or fungal biodiversity indexes when comparing net changes from T0 to T1 between control and after *B. amyloliquefaciens* based product application (**Figure 1C**). Finally, no significant changes were observed in network properties due to treatment application (data not shown).

### 3.2 Yield associated with microbiome network properties

**Figure 2:**
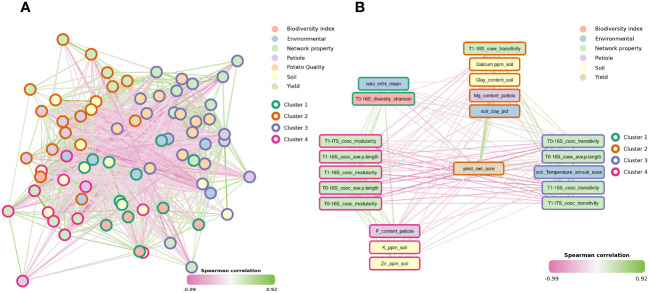
Spearman correlation network of microbiome indexes, environmental and physicochemical data from soil, leaf petiole and potato peel **(A)**. Only significant correlations are shown (p-value < 0.05). Each edge represents a Spearman correlation between two variables, while nodes represent the different variables. Nodes are coloured by variable type and borders represent the clusters assigned using Louvain clustering on the positive-edge only network. Subgraph showing only the direct neighborhood of yield **(B)**.

The global correlation network across soil, leaf petiole and peel physico-chemical properties, microbiome indexes and yield separated in four main clusters containing variables that significantly correlated (p-value threshold < 0.05) (**Supplementary Table S4**). Here, both treated and untreated samples were used to provide a global overview of the relations between microbiome, yield and physico-chemical properties. Cluster 1 mainly comprised biodiversity related variables. Cluster 2 comprised yield, specific network and leaf petiole properties, such as T0 16S co-exclusion transitivity, T0 ITS co-exclusion average path length, T0 ITS co-exclusion modularity, T0 ITS co-exclusion transitivity, calcium, nitrogen, sulfur content, and iron (**Figure 2A**; **Supplementary Table S4**). Cluster 3 included mainly potato peel and specific network properties, such as T0 16S co-exclusion modularity, T0 16S co-occurrence transitivity and T0 16S co-exclusion average path length, T1 16S co-exclusion modularity, and T1 16S co-exclusion transitivity (**Supplementary Table S4**). Finally, cluster 4 included network properties and soil properties such as T0 16S co-occurrence average path length, T1 16S co-occurrence average path length, T1 ITS co-occurrence modularity, sulfur, zinc, boron (**Supplementary Table S4**).

Notably, yield positively correlated with soil calcium, soil clay, soil clay percent from selected environmental features (see **Supplementary Table S2**) and leaf petiole magnesium content (pairwise Spearman correlation: R = 0.44, p-value < 0.001, R = 0.51, p-value < 0.001, R = 0.28, p-value = 0.034, R = 0.41, p-value = 0.001, respectively). Similarly, yield positively correlated with T0 16S diversity Shannon and T0 16S co-occurrence transitivity (pairwise Spearman correlation: R = 0.27, p-value = 0.046; R = 0.41, p-value = 0.01). Moreover, both bacterial and fungal co-occurrence transitivity positively correlated with yield, both in T1 (pairwise Spearman correlation: R = 0.42, p-value = 0.001, R = 0.44, p-value < 0.001) (**Figure 2B**). On the other hand, yield negatively correlated with bacterial co-occurrence modularity in T0 (pairwise Spearman correlation: R = -0.54, p-value < 0.001). Similarly, yield negatively correlated with both bacterial and fungal co-occurrence modularity in T1 (pairwise Spearman correlation: R = -0.48, p-value <0.001, R = -0.40, p-value = 0.002, respectively). Likewise, yield negatively correlated with bacterial co-occurrence average path length in T0 (pairwise Spearman correlation: R = -0.43, p-value < 0.001), petiole phosphorus content (pairwise Spearman correlation: R = -0.45, p-value < 0.001), NDVI of the first third of April (m04), soil potassium and zinc content (pairwise Spearman correlation: R = -0.53, p-value < 0.001, R = -0.39, p-value = 0.003, R = -0.35, p-value = 0.008).

Yield and potato quality were not correlated with each other (**Figure 2B**). Potato peel manganese positively correlated with leaf petiole manganese (pairwise Spearman correlation: R = 0.31, p-value = 0.007). Similarly, potato peel manganese positively correlated with precipitation of the wettest quarter (pairwise Spearman correlation: R = 0.28, p-value = 0.011) and NDVI m07 which is a proxy of yield (pairwise Spearman correlation: R = 0.27, p-value = 0.009) (**Supplementary Figure S5**). In addition, potato peel manganese showed associations with fungal direct neighbor nodes (**Supplementary Figure S5**). More specifically, peel manganese positively correlated with both T0 and bacterial Shannon diversity (pairwise Spearman correlation: R = 0.23, p-value = 0.04) and T1 bacterial Shannon diversity and Chao1 (pairwise Spearman correlation: R = 0.25, p-value = 0.03, R = 0.46, p-value < 0.001). On the other hand, peel manganese concentration in potato peel negatively correlated with T0 fungal Shannon diversity.

Moreover, positive correlation through pairwise Spearman correlations (p-value threshold < 0.05) were found between potato peel zinc and abiotic and biotic soil characteristic. For instance, it positively correlated with soil sand content, soil temperature of the wettest quarter, T1 ITS co-occurrence transitivity. Moreover, it positively correlated with main potato peel properties such as calcium, iron, copper and manganese (**Supplementary Figure S6**). Finally, by exploring individual correlations between microbiome indexes and metadata, some correlations were detected to possibly be location driven (**Supplementary Figure S7**). For instance, the correlation between bacterial and fungal Shannon diversity and Chao1 with soil phosphorus, organic matter and leaf petiole zinc seemed to be driven by Loc16. Similarly, the correlation between bacterial and fungal Shannon diversity and Chao1 and soil calcium seemed to be driven by Loc1 and Loc20 (**Supplementary Figure S7A**). The correlation with phosphorus content was driven by Loc9 and Loc11 (**Supplementary Figure S7B**), this tendency was not observed for potato quality properties (**Supplementary Figure S7C**).

### 3.3 Application of the *B. amyloliquefaciens* based product significantly impacts fungal microbiome composition

**Figure 3:**
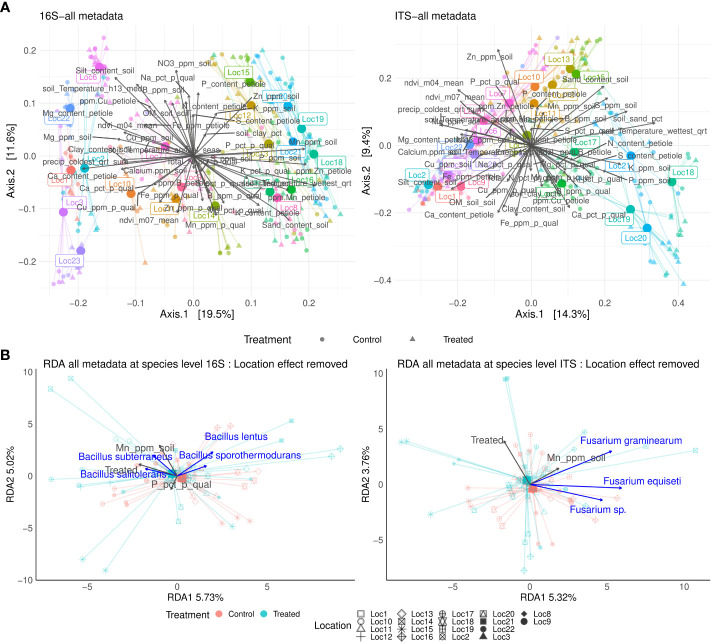
Principal coordinate analysis (PCoA) of the microbial community based on Bray-Curtis distances for 16S and ITS markers at global scale. Correlation with soil, leaf petiole, potato peel nutrients to microbiome ordination are shown for each marker **(A)**. Constrained ordination for 16S and ITS community composition fitting treatment, soil, leaf petiole, potato peel and environmental features. Location effect was removed. Only significant terms (p-value < 0.05) are reported **(B)**.

Beta diversity analysis showed that location played a major role in determining soil microbiome composition (Permanova: 16S F_[20]_ = 21.76, R = 45.46%; ITS F_[20]_ = 13.59, R = 45.15%, p-value <0.001) followed by its interaction with treatment (16S F_[1,20]_ = 1.63, R = 3.71%; ITS F_[1,20]_ = 1.39, R = 4.61%, p-value <0.001 (location: treatment) (**Figure 3A**; **Table 1**). Therefore, the interaction location:treatment:time significantly explained microbiome composition variation depending on the location, especially for fungal community (F_[1,20]_ = 1.52, R = 3.47%, p-value <0.001 Bacteria, F_[1,20]_ = 1.39, R = 4.61%, p-value <0.001 fungi (**Figure 3A**; **Table 1**). Notably, silt content and soil manganese correlated with bacterial microbiome ordination (R = 45.55%, p-value = 0.01; R = 39.93%, p-value=0.01), while soil zinc and sand contents correlated with fungal ordination (R = 38.27%, p-value = 0.01; R = 33.38%, p-value = 0.01) (**Supplementary Table S5**). Moreover, soil temperature of the wettest quarter correlated with both bacterial and fungal microbiome ordination (R = 51.14%, p-value = 0.01; R = 40.37%, p-value = 0.01), while precipitation only with bacterial microbiome ordination (R = 40.23%, p-value = 0.01) (**Supplementary Table S5**). Correlation of all soil, leaf petiole nutrients, potato quality and environmental variables can be found in **Supplementary Table S6**.

**Table 1:** PERMANOVA analysis on microbiome ordination for time, treatment and location factors.

Marker	Term	Variance explained	p-value
16S	Location	49.45%	0.0001
	Time	2.48%	0.0001
	Location : Treatment	3.71%	0.0001
	Location : Time	20.38%	0.0001
	Location : Treatment:Time	3.47%	0.0002
ITS	Location	45.15%	0.0001
	Time	1.60%	0.0001
	Location : Treatment	4.61%	0.0001
	Location : Time	14.11%	0.0001
	Location : Treatment:Time	4.61%	0.0001

Significant terms (p-value < 0.05) and their variance explained by marker.

Next, constrained microbiome ordination by location using the above-mentioned variables was performed to remove the location effect. Application of the *B. amyloliquefaciens* explained the significant changes in the fungal composition, (**Table 2**, **Figure 3B**), but not in bacterial composition. In fact, potential plant associated pathogens, such as *Fusarium* species (e.g. *Fusarium* sp., *F. graminearum* and *F. equiseti*) tended to have positive association with control samples. (**Figure 3B**). Regarding nutrients, phosphorus content in potato peel significantly modulated bacterial composition variation. Conversely, soil manganese was the only meaningful physico-chemical factor to explain fungal composition variation (**Table 2**).

**Table 2:** RDA analysis significant model terms (p-value< 0.05) and their explained variance.

Marker	Term	Variance explained	p-value
16S	P pct	2.07%	0.046
ITS	Treatment	2.05%	0.01
	Mn ppm	2.21%	0.007

### 3.4 Yield directly associated with specific microbial taxa

**Figure 4:**
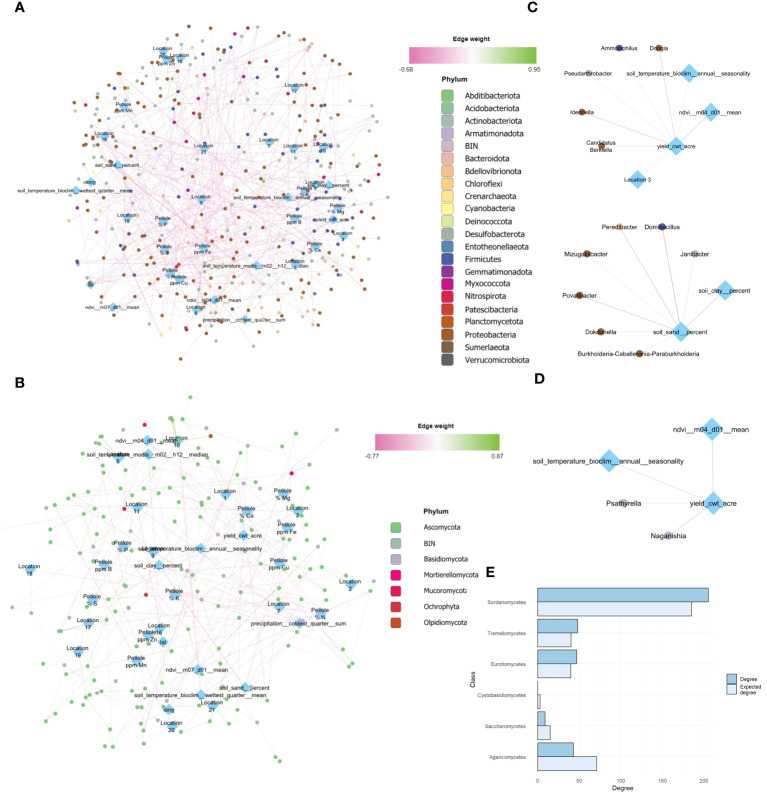
Global network analysis at genus taxa level after MINUET™ application, introducing metadata variables (yield, climate and petiole, peel physicochemical properties). Metadata variables are annotated in blue rhomboid nodes, while taxa is annotated in circular nodes colored by the Phylum they belong to. Connections among nodes are represented by positive or negative edges, ranging from green to pink, respectively. Global network for bacterial community **(A)**. Global network for fungal community **(B)**. Direct neighbors of relevant metadata variables, such as yield for bacterial **(C)** and fungal network **(D)**. Difference between observed and expected degree for the six fungal classes with the largest positive or negative difference in degree in the ITS network. Classes with a higher degree than expected are shown above, and those with a lower degree than expected are below **(E)**.

Yield was highly and positively associated with several bacterial taxa, such as *Pseudoarthrobacter* (FlashWeave association weight: 0.34) and, *Ammoniphilus* (+) (FlashWeave association weight: 0.34), but negatively with *Ideonella, Candidatus Berkiella, Dongia* (-) (FlashWeave association weight: -0.38 -0.30, -0.36, respectively) (**Figures 4A–C**). Moreover, yield associated with fungal taxa such as *Psathyrella and Naganishia* (FlashWeave association weight: 0.36, -0.35, respectively) (**Figure 4D**). *B. amyloliquefaciens* based product had no significant associations at taxa level, but slighter effects might be blurred if those effects can be explained by other nodes in the network (**Figure 4A**). On the other hand, NDVI of the month of April (m04) was negatively associated with yield (FlashWeave association weight: -0.35), as shown by its central position in the network (**Figure 4C**).

In the bacterial network, the phyla Proteobacteria and Actinobacteriota were responsible for most associations; the total degree (connectance of each taxa) for these groups was 467 and 293 respectively, compared to 148 for Firmicutes, the third most-connected phylum (**Figure 4A**). Additionally, the degree and betweenness centrality was strongly correlated for both 16S and ITS networks (p < 0.001, and respectively). However, the total degree for both Proteobacteria and Actinobacteria was lower than expected. On the other hand, Acidobacteriota, Firmicutes and Planctomycetota had a total degree at least 10 greater than the expected total degree.

Finally, for the fungal global network, Ascomycota played a larger role in the fungi community structure, followed by Basidiomycota (green nodes) (**Figures 4B, C**, green nodes). Moreover, soil temperature, precipitation and sand percentage occupied central positions, but NDVI was relatively more connected in the ITS network (**Figures 4B, C**). Ascomycota had a higher total degree than expected, while Basidiomycota had a lower total degree than expected. Here, the classes Sordariomycetes, Tremellomycetes and Eurotiomycetes had a higher total degree relative to the expected degree (**Figure 4B**).

### 3.5 *B. amyloliquefaciens* based product impacts preserved and accessory microbiome fraction

**Figure 5:**
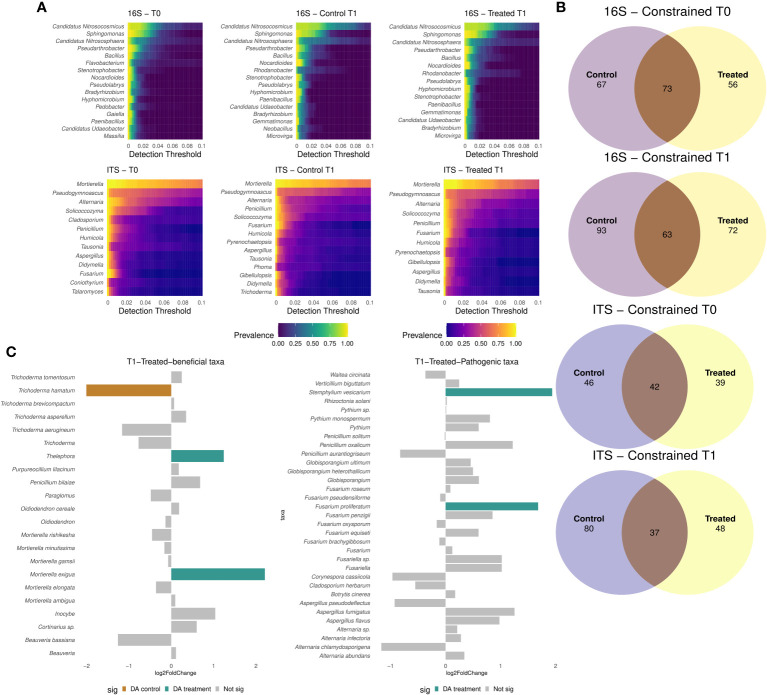
Heatmaps showing the genus prevalence proportion across different detection thresholds for 16S and ITS markers **(A)** in combined treated and control samples at T0 and control and treated samples in T1 **(A)**. Constrained shared and exclusive members of bacterial and fungal microbiomes at Genus level, for 16S and ITS, respectively **(B)**. Barplot showing potential beneficial differential abundant taxa **(C)** and potential pathogenic taxa **(C)** in treated samples at T1 when compared to the control reporting their log fold change.

In order to identify core microbiome members, only taxa found in all locations with a detection threshold of 0.01 and a prevalence of 25% were considered for the analysis. The preserved microbial fraction tended to be 25% higher, when comparing the preserved and accessory microbiome fraction of bacterial and fungal communities in T0 and in untreated T1, vs samples treated with *B. amyloliquefaciens* in T1. A more conserved fraction of bacterial genera was detected compared to fungal genera (**Figure 5A**). *Candidatus*, *Nitrosocosmicus* and *Sphingomonas* core members were higher in samples treated with the *B. amyloliquefaciens* based product, in comparison to the control at T1 (**Figure 5A**). No major differences were detected in the bacterial core microbiome between control and treated samples. Regarding fungal microbiome, *Mortierella* was the only fungal genus with a global prevalence higher than 80% (**Figure 5A**). Notably, *Fusarium* and *Trichoderma* were present in the control core microbiome at higher prevalence (**Figure 5A**). In order to determine core microbiome size, genera found in all locations were considered. Core size number of taxa decreased in all samples at T1 when compared to their T0, for both communities (**Figure 5B**). Moreover, the shared number of genera between control and treated samples was lower in T1 compared to T0 (**Figure 5B**). None of the bacterial taxa were differentially abundant (Wald test: pval > 0.05) due to application of the *B. amyloliquefaciens* based product. However, application of *B. amyloliquefaciens* based product impacted the abundance of several fungal taxa. For instance, *Mortierella* increased in relative abundance after treatment with the *B. amyloliquefaciens* based product, while *Stemphylium* and *F. proliferatum* increased after treatment with *B. amyloliquefaciens* (**Figure 5C**; **Supplementary Figure S9**).

## 4 Discussion

In this work, the effects of *B. amyloliquefaciens* based product on soil microbiome were further explored across 21 locations from the United States integrating yield, soil, leaf petiole nutrients, potato peel quality and environmental data.

Yield and leaf petiole boron showed a significant increase after treatment with *B. amyloliquefaciens* based product. Our results on yield are in agreement with the previous study applying *B. amyloliquefaciens* QST713 in the United States (Imam et al., 2021). Imam et al. (2021) observed a significant yield increase after inoculation of *B. amyloliquefaciens* QST713 in two geographical locations, now expanded to more USA locations. In addition, previous studies already assessed the importance of boron for plant growth (Tombuloglu et al., 2017; Pereira et al., 2021). Boron is involved in cell elongation, nucleic acid synthesis, hormone responses and membrane function (Pereira et al., 2021) and its plant absorption capacity increases with higher soil clay content (Padbhushan and Kumar, 2017). Our results showed a significant positive association with soil clay content. This is well known to be an important factor for potato productivity, since its fine texture prevents nutrient leaching and enhances water availability (Zebarth et al., 2021). Therefore, the enhancement of yield and boron after product inoculation in these soil conditions highlights the indirect role that *B. amyloliquefaciens* may have in nutrient mobilization and yield improvement (Rana et al., 2012).

Moreover, potato peel manganese, phosphorus and zinc content significantly increased after *B. amyloliquefaciens* inoculation. Our results are in line with previous findings, where increased concentrations of the above nutrients improved after rhizobacteria inoculation (Ipek et al., 2014). The increased concentration of peel phosphorus after inoculation may be due to the bacterial solubilization of P, increasing its availability in the soil for the plant (Rouphael and Colla, 2020).

Focusing on different geographic regions, yield, bacterial and fungal richness and evenness significantly changed across locations independently of time. These results confirm that geographical location is one of the main drivers of potato yields and soil microbiome, due to edaphic and climate variation of the different regions (Rasche et al., 2006; Weinert et al., 2010). In addition, bacterial and fungal communities were mainly explained by location, followed by its interaction with treatment. The impact of geographical location in the bacterial communities was slightly higher than for the fungal communities. This was also evidenced when correlating geographical distances with beta diversity distance matrix (**Supplementary Figure S8**). Moreover, our results showed that treatment with *B. amyloliquefaciens* based product biological fungicide impacted soil microbiome communities depending on the location, especially for the fungal community (see **Table 1**). This indicates that the treatment may impact soil microbiome differently due to the climatic and edaphic conditions, which might potentially further explain the different nutrient mobilization effect in different geographical locations. Previous studies already assessed biogeographical patterns as the main drivers of soil microbiome (Hanson et al., 2012; Bahram et al., 2013; Tedersoo et al., 2014; Plassart et al., 2019).

The biological product had no significant impact on either bacterial or fungal alpha-diversity. Similarly, Imam et al. (2021) detected no changes in either bacterial or fungal alpha diversity in any location, even if they observed shifts in soil microbial structure (Imam et al., 2021). However, the application of a plant growth promoting bacteria did not affect the soil bacterial community structure in maize (Kari et al., 2021). These observations were explained by possible resilience of the ecosystem, driven by the interactions between plant and soil microbes. Nevertheless, yield had a significant positive associations with both bacterial and fungal co-occurrence transitivity, while negative associations with modularity. This indicates that communities that are more interconnected (increased transitivity) may lead to higher yields. On the other hand, a highly modular microbiome (increased modularity), where taxa form specific niches, may lead to lower yields. Therefore, both bacterial and fungal local network properties may be good drivers of yields due to their strong correlation profile, as similarly shown in previous studies (Imam et al., 2021). In conclusion, preserving microbial community structure where communities are more interconnected and cooperative, may be an important target to achieve higher crop performance.

Notably, yield was positively correlated with soil calcium. These results are in agreement with previous studies that assessed the important role of calcium for potato productivity (Gondwe et al., 2019; Koch et al., 2020). Calcium is a fundamental micronutrient that enhances plant growth and is a signaling molecule mediating plant response to environmental stresses and hormones (Thor, 2019). However, the negative correlation with the NDVI during the month of April indicates that lower NDVI after planting season may not be predictive of yields. In fact, higher yields at the harvesting season were associated with lower NDVI in the month of April.

Moreover, treatment with *B. amyloliquefaciens* based product had a significant effect on fungal community composition at T1 but not on bacterial community. These results are in agreement with previous studies, where the inoculation of *B. amyloliquefaciens* BNM122 in soybean did not significantly change the bacterial community of the rhizosphere (Correa et al., 2009). Similarly, in potato, the inoculation of *B. subtilis* did not significantly affect the bacterial or fungal community, while improving potato yield (Song et al., 2021). Conversely, the inoculation of *Stenotrophomonas rhizophila* indirectly promoted plant growth by shaping the soil fungal community in tomato and sweet peppers (Schmidt et al., 2012). In addition, Fusarium species such as *Fusarium* sp. and *F. equiseti* tended to be associated with control samples. Therefore, potential plant pathogens may have reduced presence when *B. amyloliquefaciens* is applied.

The efficacy of *B. subtilis* against *Fusarium* was already observed in potato cultivars (Gachango et al., 2012; Stefańczyk et al., 2016; Tiwari et al., 2020). In particular, the inoculation of *B. subtillis* in potato crops effectively reduced *Fusarium* spp. abundance, including *F. graminareum* (Khedher et al., 2021). Similarly, a previous study in apple showed that *B. amyloliquefaciens* QSB-6 inhibited several *Fusarium* species, while significantly improving seedling growth (Duan et al., 2021). Nevertheless, further studies for different potato genotypes are needed to assess the pathogenicity of *Fusarium* spp. and possible biocontrol action of *B. amyloliquefaciens* against it. Conversely, *B. amyloliquefaciens* based product promoted the potential pathogen *F. proliferatum* (Gachango et al., 2012). Therefore, treatment with the *B. amyloliquefaciens* based product may have a greater impact on specific *Fusarium* taxa. Notably, soil sand content significantly explained fungal community composition variation.

At taxa level, the *B. amyloliquefaciens* based biological fungicide had no direct association with bacteria and fungi network when taxa were included in the global network integrating yield and petiole nutrients. However, weaker or indirect treatment effects on community structure may not be detected if those effects can be explained by other nodes in the network. Nevertheless, yield had strong associations with several bacterial taxa, such as positive associations with *Pseudoarthrobacter* (FlashWeave association weight: 0.34) and *Ammoniphilus* (+) (FlashWeave association weight: 0.34), and negative with *Ideonella, Candidatus Berkiella, Dongia* (-) (FlashWeave association weight: -0.38 -0.30, -0.36, respectively). A recent study assessed the effects of the inoculation of chlorophenolicus in *Geum aleppicum*, which showed a significant increase in root development and plant growth (Ham et al., 2022). Therefore, *Pseudarthrobacter* may promote potato yield, through the stimulation of root development. However, further studies should be carried out on rhizosphere targeting potato crop and *Pseudarthrobacter*. In addition, several uncultured taxa are involved in nitrogen fixation, which could promote N availability to the plant (Mus et al., 2016). Regarding relevant taxa, Proteobacteria and Actinobacteriota were responsible for most associations. Notably, these taxa have a fundamental role in Nitrogen cycling (Kielak et al., 2016; Mosley et al., 2022). and may promote nitrogen availability. In addition, Firmicutes and Planctomycetota had a total degree of prevalence in the network at least 10 units higher than the expected total degree. This indicates that Firmicutes and Planctomycetota occupy more central positions in the network, highlighting their importance on community structure. *Bacillus* a is well-known beneficial taxon for soil health (Saxena et al., 2020). Hence, favoring *B. amyloliquefaciens* presence through inoculation may lead to better connected networks due to Firmicutes high degree and central role. For the ITS network, Ascomycota had a higher total degree than expected compared to Basidiomycota, suggesting this phylum played a larger role in the community structure. In particular, the class Sordariomycetes includes many potential saprotrophic taxa fundamental for crop litter decomposition (Wang et al., 2021). Moreover, species belonging to the Sordariomycetes may be able to grow in fecal material and they can be an indicator of differences in fertilization application between locations (Guo et al., 2022).

Lastly, *B. amyloliquefaciens* biological fungicide slightly modulated the soil core microbiome from T0 to T1. In particular, a higher conserved fraction of *Sphingomonas sp* was observed. A recent study on maize showed that *Sphingomonas* sp. Hbc-6 increased microbiome rhizosphere diversity and could help plant growth promotion by recruiting beneficial bacteria in inoculated soils (Wang et al., 2022). Conversely, genus *Fusarium* in treated condition showed a lower detection threshold in T1, when compared to control at T1. In addition, no changes in the detection threshold were seen for the genus Mortierella between conditions. *Mortierella* is a widely spread genus which is known to be beneficial in soils (Ozimek and Hanaka, 2020). Therefore, treatment with *B. amyloliquefaciens* may modulate the presence of potential pathogens such as *Fusarium*, while indirectly promoting beneficial taxa through indirect effect (Cao et al., 2011; Khedher et al., 2021). Moreover, species like *Stemphylium* and *F. proliferatum* were differentially abundant after treatment with *B. amyloliquefaciens*. Therefore, these species may be more resistant to the product and a better competitor than other taxa. Hence, these taxa may increase after treatment application while other taxa decrease. However, further analyses should be performed to decipher the effects that treatment with *B. amyloliquefaciens* can have on different *Fusarium* species.

Finally, our results showed that soil inoculation with a sustainable *Bacillus*-based product correlated with higher yields. In addition, nutrient solubilization and soil health were promoted, without major disruption of the soil microbiome. Indeed, these findings contribute to disentangle sustainable long term solutions in view of the future global climate change, increasing global food demand.

## 5 Conclusions

Our results showed that treatment with *B. amyloliquefaciens* based product correlated with enhanced yield. In addition, the treatment was associated with leaf petiole boron and improved nutrient uptake in potato peel. Therefore, *B. amyloliquefaciens* based product may indirectly promote nutrient solubilization with minor impacts on the soil microbiome diversity 30 days after inoculation. Moreover, yield was strongly correlated to specific local network properties, which are associated with cohesive and cooperative microbial community. This highlights the importance of the native soil microbiome with a complex and interconnected structure, indirectly promoting healthier soils. In addition, treatment with *B. amyloliquefaciens* had an impact on fungal community composition, reducing *Fusarium* presence without major impacts on the fungal community structure. Moreover, the *B. amyloliquefaciens* based product modulated the soil core microbiome after 30 days by modulating the presence of *Fusarium*, while indirectly promoting beneficial taxa like *Sphingomonas* and *Mortierella*. However, further studies should focus on the long term effects of *B. amyloliquefaciens* based product on soil bacterial and fungal communities. Lastly, our results showed that the use of a sustainable biostimulant product correlated with higher yield and indirectly promoted soil health. These evidences are crucial to further enhance agriculture sustainability, increasing food production while reducing’s environmental footprint.

## Data availability statement

The datasets presented in this study can be found in online repositories. The names of the repository/repositories and accession number(s) can be found below: https://www.ncbi.nlm.nih.gov/, PRJNA981118.

## Author contributions

IA: Data curation, Formal analysis, Investigation, Resources, Software, Visualization, Writing – original draft. MA-A: Conceptualization, Data curation, Formal analysis, Investigation, Methodology, Software, Validation, Visualization, Writing – original draft. SR: Data curation, Investigation, Software, Validation, Writing – review & editing. DP: Data curation, Investigation, Software, Validation, Writing – review & editing. BB: Data curation, Writing – review & editing. JZ: Data curation, Writing – review & editing. RG: Conceptualization, Methodology, Writing – review & editing. BG-J: Conceptualization, Data curation, Methodology, Supervision, Validation, Writing – original draft, Writing – review & editing. PJ-R: Conceptualization, Data curation, Methodology, Resources, Supervision, Writing – review & editing. LC: Conceptualization, Data curation, Methodology, Project administration, Resources, Writing – review & editing. AA: Conceptualization, Methodology, Project administration, Supervision, Writing – review & editing.
