# Supplementary material for: Short impact on soil microbiome of a Bacillus amyloliquefaciens QST713 based product that correlates with higher potato yield across USA
Source: Front Plant Sci. 2024 Mar 12;15:1332840. doi: 10.3389/fpls.2024.1332840 (PMC10967024; doi:10.3389/fpls.2024.1332840)
Supplement: Supplementary file 1 [file Image_1.pdf]

Supplementary Material

1 SUPPLEMENTARY TABLES AND FIGURES

1.1 Figures

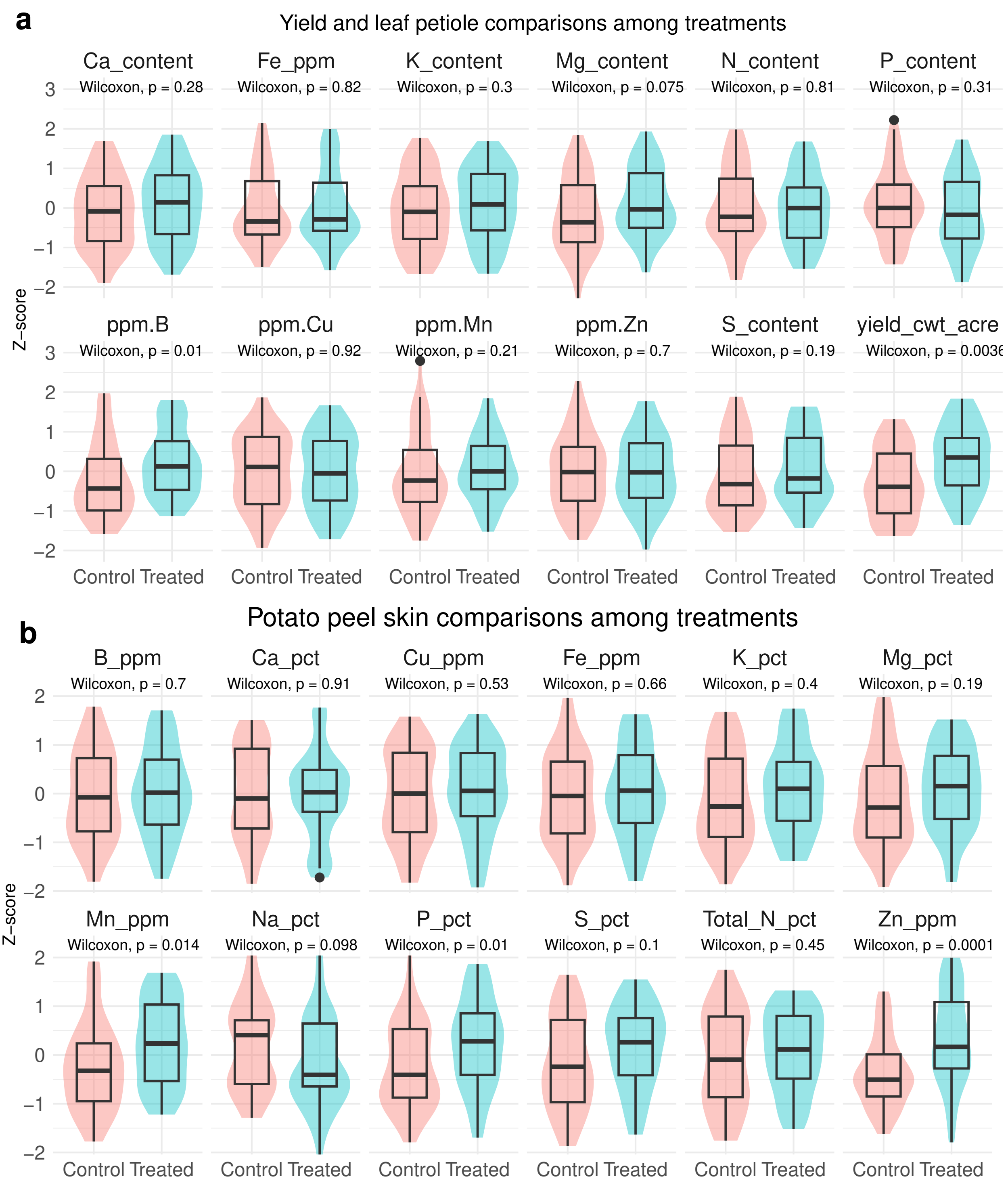

Figure S1: Leaf Petiole and yield (panel a) and potato peel (panel b) comparison between treatments and control normalized per location.

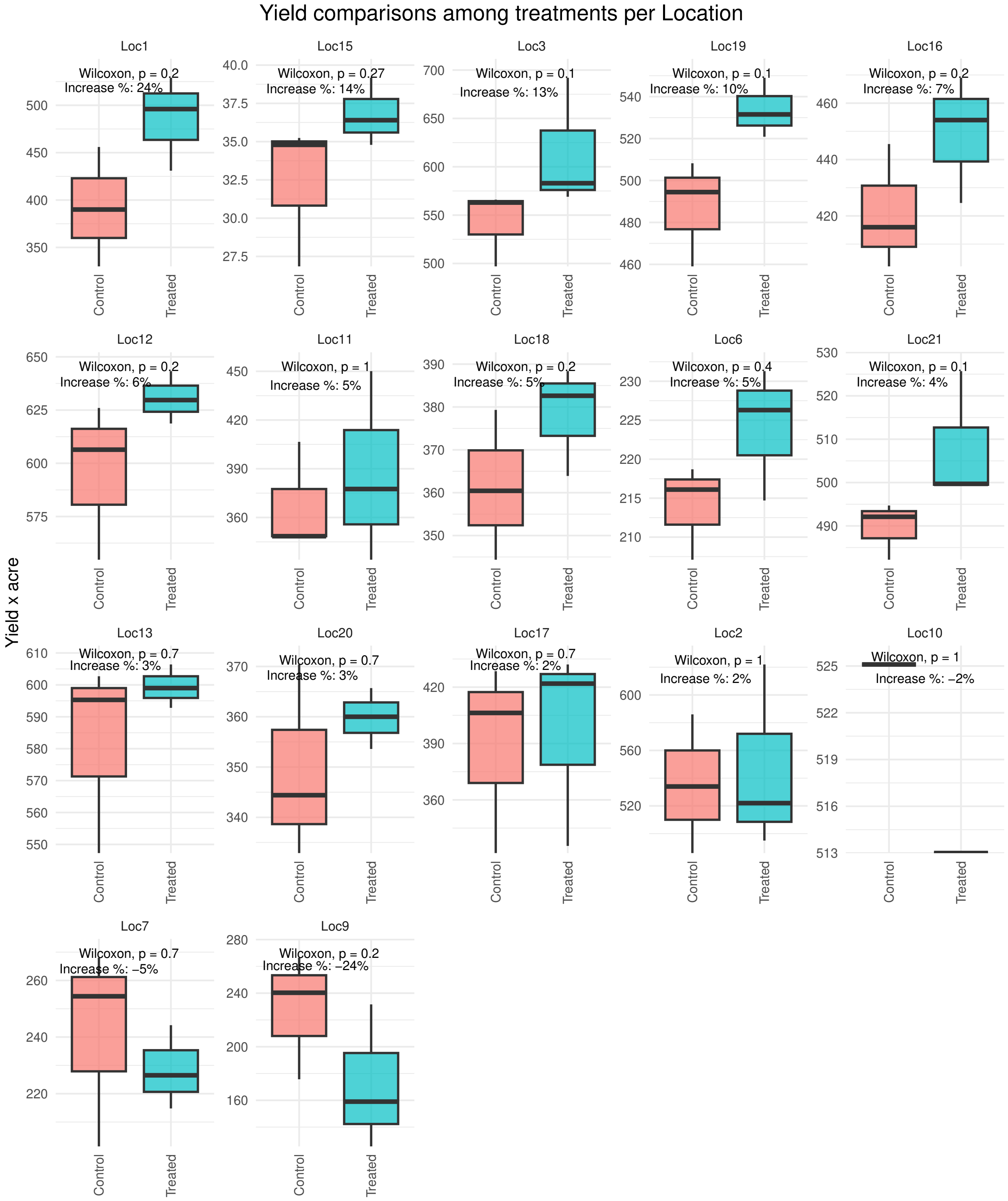

Figure S2: Per location raw yield comparisons among treatments. Percentage of increase was calculated for each location, with positive values indicating a positive yield increase from control to treated. Note that axis Y has different scales

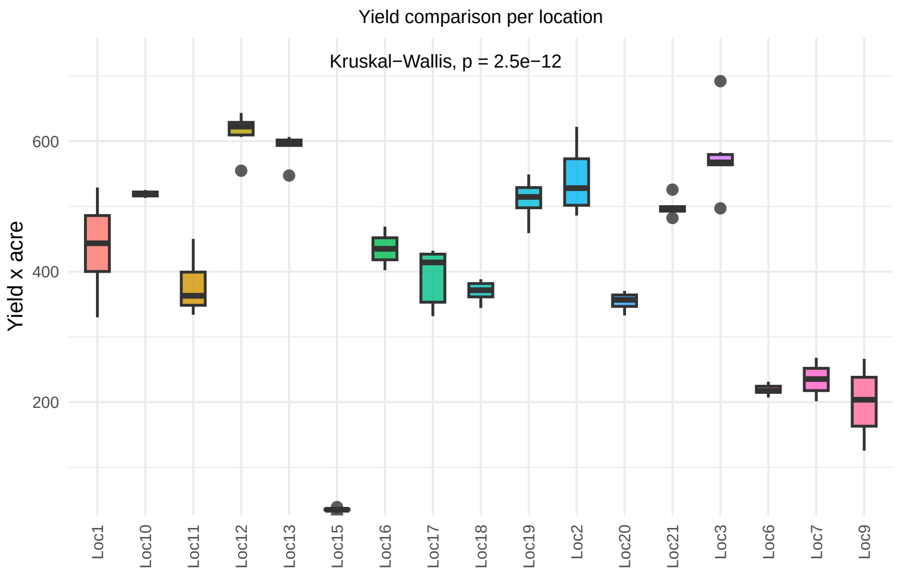

Figure S3: Overall raw yield per location comparison (treatment and control)

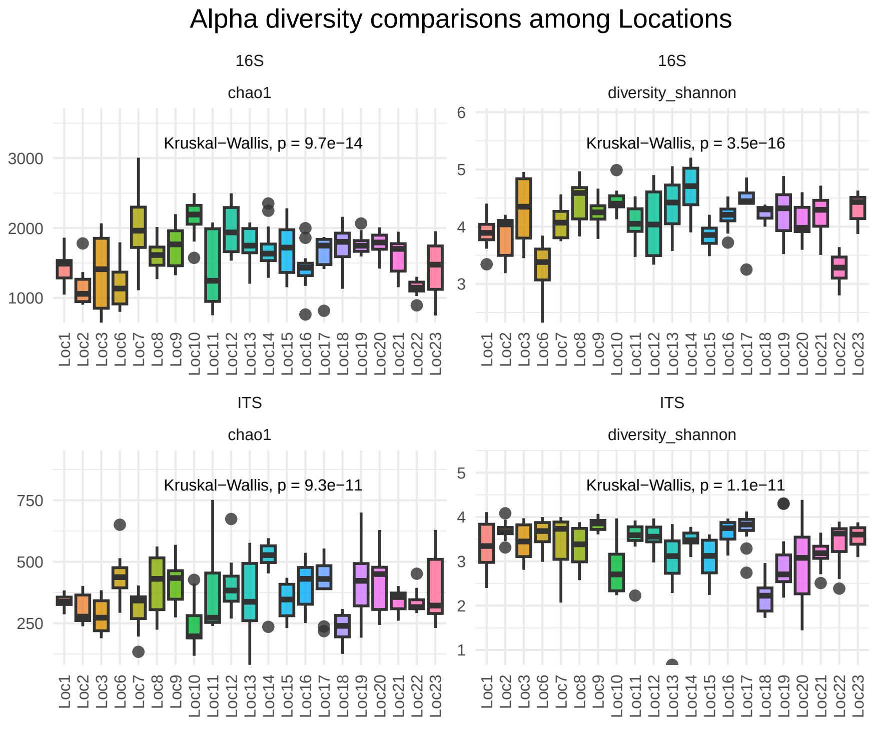

Figure S4: Overall biodiversity comparison among locations

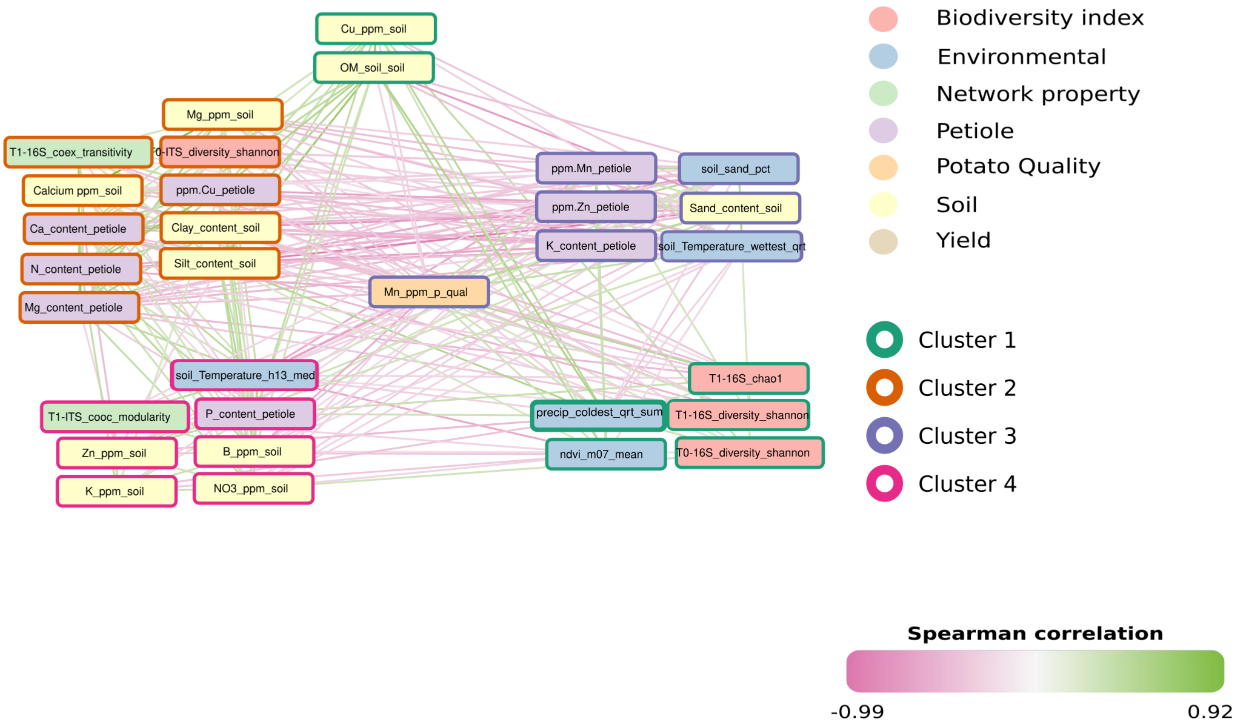

Figure S5: Subgraph showing only the neighborhood of potato peel Manganese. Each edge represents a Spearman correlation between two variables.

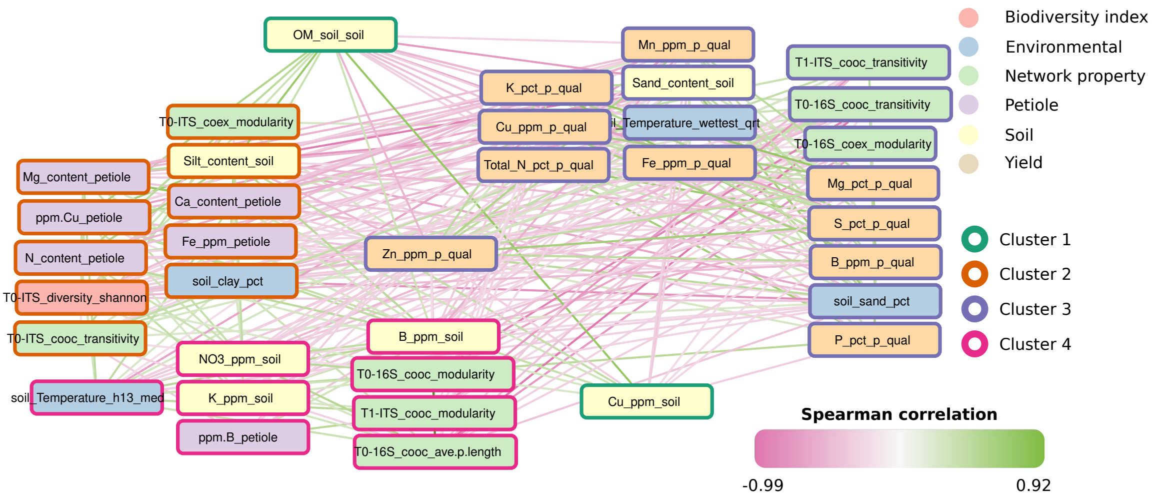

Figure S6: Subgraph showing only the neighborhood of potato peel Zinc. Each edge represents a Spearman correlation between two variables.

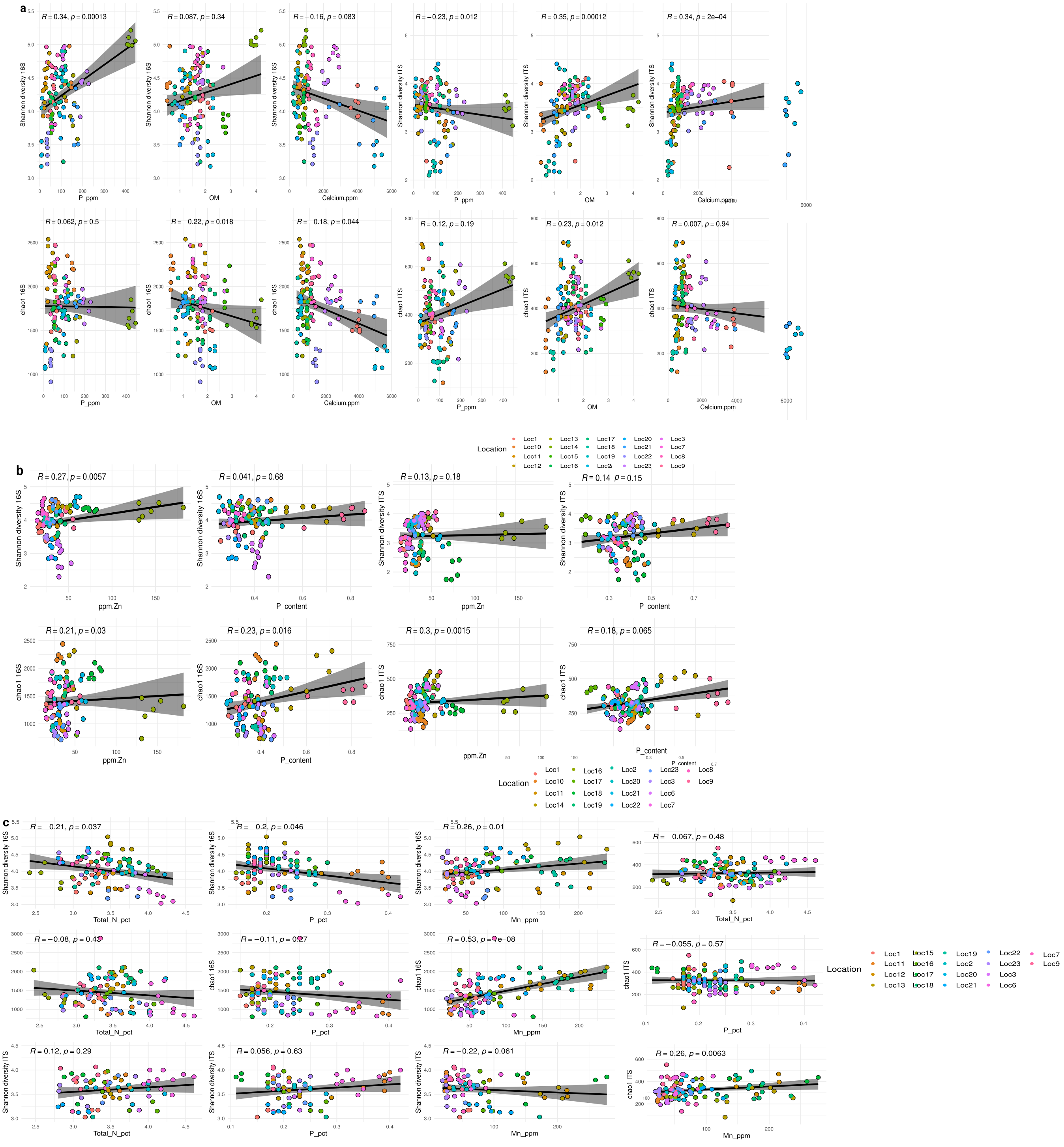

Figure S7: Scatter plots of alpha diversity indexes and soil (panel a), petiole (panel b) and potato peel data (panel c).

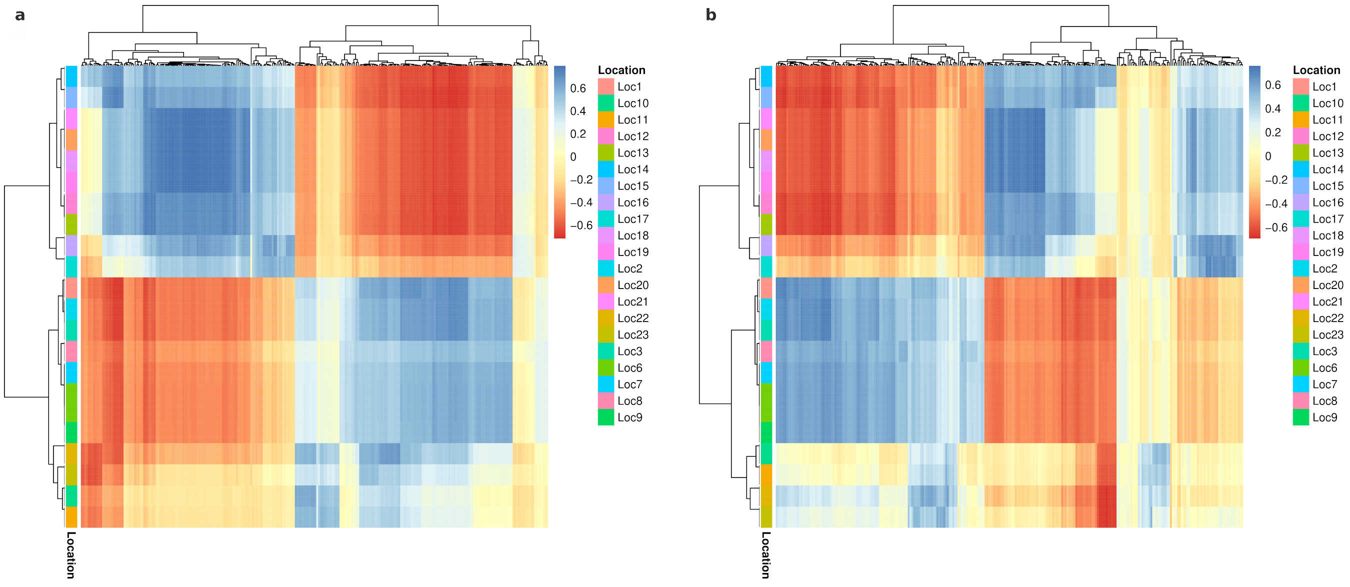

Figure S8: Correlation clustering of geographical and beta diversity indexes for 16S (left) and ITS (right) markers

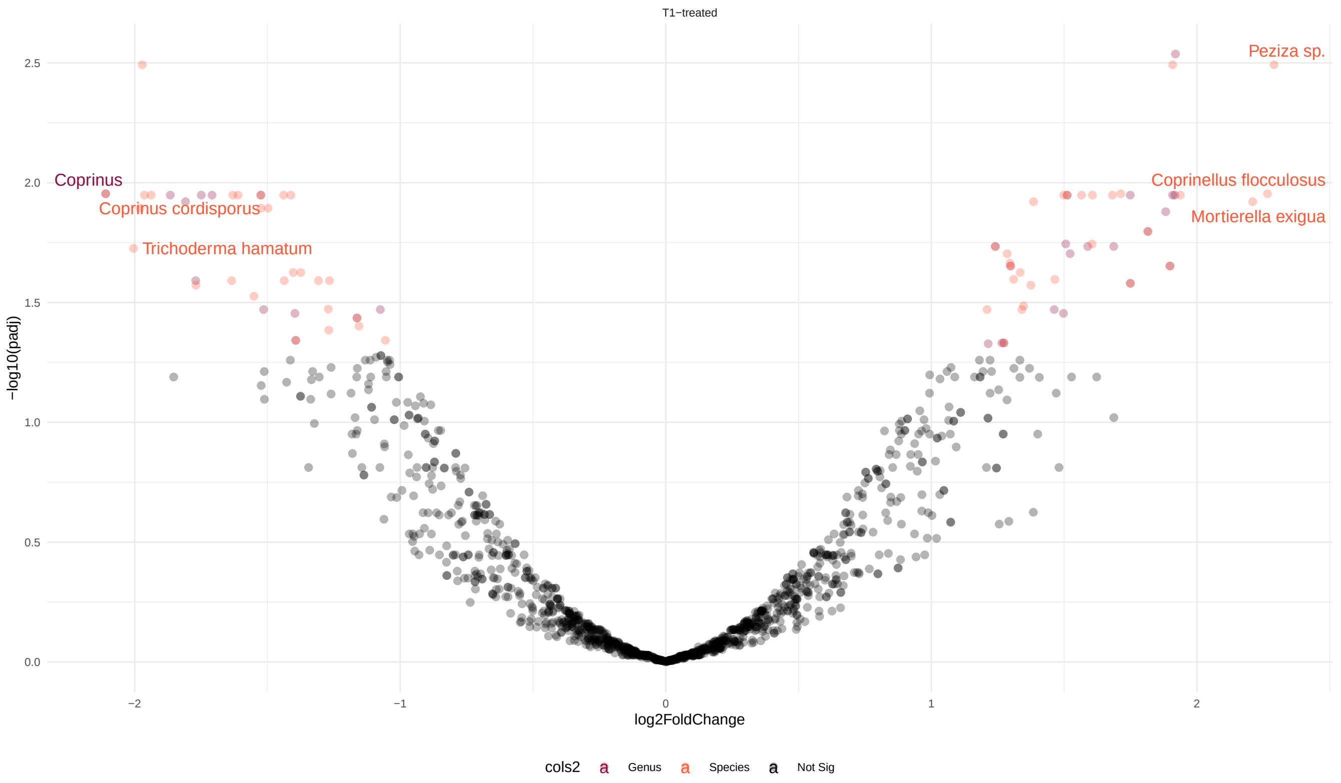

Figure S9: Volcano plot showing differentially abundant (abs(log2Fold change) greater than 2 and adjusted pval smaller than 0.05 at different taxonomic levels using negative binomial distribution (DESeq2) at T1 treated compared to T0.
